# Loss of Toll-like receptor 7 alters cytokine production and protects against experimental cerebral malaria

**DOI:** 10.1186/1475-2875-13-354

**Published:** 2014-09-05

**Authors:** Alyssa Baccarella, Brian W Huang, Mary F Fontana, Charles C Kim

**Affiliations:** Division of Experimental Medicine, Department of Medicine, University of California, San Francisco, CA 94143 USA

**Keywords:** Cerebral malaria, *Plasmodium berghei*, Toll-like receptors, TLR7, Cytokines, Mouse

## Abstract

**Background:**

Malaria, caused by *Plasmodium sp.* parasites, is a leading cause of global morbidity and mortality. Cerebral malaria, characterized by neurological symptoms, is a life-threatening complication of malaria affecting over 500,000 young children in Africa every year. Because of the prevalence and severity of cerebral malaria, a better understanding of the underlying molecular mechanisms of its pathology is desirable and could inform future development of therapeutics. This study sought to clarify the role of Toll-like receptors (TLRs) in promoting immunopathology associated with cerebral malaria, with a particular focus on the understudied TLR7.

**Methods:**

Using the *Plasmodium berghei* ANKA mouse model of experimental cerebral malaria, C57BL/6 mice deficient in various TLRs were infected, and their resistance to cerebral malaria and immune activation through cytokine production were measured.

**Results:**

Loss of TLR7 conferred partial protection against fatal experimental cerebral malaria. Additionally, loss of TLR signalling dysregulated the cytokine profile, resulting in a shift in the cytokine balance towards those with more anti-inflammatory properties.

**Conclusion:**

This work identifies signalling through TLR7 as a source of pathology in experimental cerebral malaria.

## Background

Malaria, caused by protozoan parasites of the genus *Plasmodium*, is a major source of global morbidity and mortality, resulting in an estimated 154–289 million infections and 660,000 deaths in 2010 [1]. Approximately 12% of fatal infections in African children are caused by cerebral malaria, a severe neurological complication of *Plasmodium falciparum* infection characterized by coma (inability to localize a painful stimulus), presence of *P. falciparum* parasites in the blood, and exclusion of other causes of encephalopathy [2]. Without treatment, cerebral malaria is nearly universally lethal; with intervention, mortality is 15-20%. Furthermore, of children who survive cerebral malaria, approximately 15% exhibit neurological sequelae, from which a proportion of children experience permanent neurological impairment [3, 4]. Although this severe form of malaria represents only a modest proportion of cases, the overall high incidence of malaria results in an estimated 575,000 cases of cerebral malaria occurring annually in Africa [5]. The large number of cases, compounded by the severity of cerebral malaria, makes a better understanding of the underlying molecular mechanisms, and therapeutics based thereon, desirable.

The nature of the pathogenesis of cerebral malaria is controversial [6–12], but is thought to involve the excessive production of pro-inflammatory cytokines [13], the accumulation of leukocytes in the brain [14], and/or the sequestration of infected erythrocytes in the microvasculature of the brain [15, 16]. In the most widely used mouse model of cerebral malaria (infection of C57BL/6 mice with *Plasmodium berghei* strain ANKA, reviewed in [17]), it is clear that the activation of pro-inflammatory mechanisms results in cerebral immunopathology and symptoms. In particular, the balance of inflammatory Type I cytokines, such as interferon gamma (IFNG) and tumour necrosis factor (TNF), with Type II cytokines, *e.g.*, interleukin 4 (IL4) and IL10, is thought to determine the lethality of cerebral malaria [18, 19]. In both humans and mouse models, high levels of TNF are correlated with cerebral malaria [20, 21]. Conversely, IL10 is thought to limit cerebral pathology in mice [22, 23], and IL10 polymorphisms are associated with cerebral malaria [24]. However, translation of these findings into anti-TNF trials in humans was unsuccessful [4, 25], highlighting the need to better understand the molecular drivers of cerebral pathology.

Toll-like receptors (TLRs) are a family of innate immune sensors that are prime candidates for initiating immune responses that promote cerebral malaria. Evidence for a TLR-dependent contribution to cerebral malaria pathology stems from the observation that mice lacking myeloid differentiation factor 88 (MYD88), a downstream adapter shared by most TLRs, are partially protected from developing cerebral malaria pathology during infection with *P. berghei* [26–29] (see [30] for dissenting evidence). There is a growing body of evidence supporting TLR recognition of a broad range of *Plasmodium* molecules [31–41]; however, controversy exists about individual receptor contributions to cerebral malaria pathology. For example, TLR2 is thought to recognize *Plasmodium* glycophosphatidylinositol [33, 39]; however, whereas studies have found that *Tlr2*^*-/-*^ mice [26] and *Tlr2*^*-/-*^*Tlr4*^*-/-*^ mice [29] escaped from initial cerebral malaria at a higher rate than wild-type mice, others have reported no differences between wild-type mice and mice lacking these sensors [28, 30, 42]. TLR9, an endosomal DNA sensor, is a more well-established sensor of both mouse and human malaria parasites [31, 32, 37, 40, 41]; however, similar to the controversy over the role of TLR2 in experimental cerebral malaria, some studies have found that TLR9 deficiency results in protection from disease [26, 28], whereas other studies have found no differences as compared with wild-type mice [30, 42]. In the studies that have reported a protective effect of TLR9 disruption, cerebral malaria was still more severe than that observed in MYD88-deficient mice, suggestive of signalling contributions from other MYD88-dependent pathways [28]. It is likely that, as with other parasitic infections, multiple TLRs contribute to the immune response [43–45], making it clear that quantitative measures of immune activation are needed to better understand the relative contributions of TLRs to immune activation during malaria infection.

Previous work from this lab showed that a deficiency in TLR7, which recognizes ribonucleic acid (RNA), leads to widespread immune dysfunction during *Plasmodium chabaudi* infection, although there was no impact on the clearance of parasites [41]. Similar results have been reported for TLR9 and the shared adapter molecule, MYD88; whereas loss of MYD88 or TLR9 diminished cytokine production during *P. chabaudi* infection, no changes in control of parasitaemia were seen [46]. Similarly, during *P. berghei* strain ANKA (referred to as ‘*P. berghei’* hereafter) infection of the C57BL/6 mouse, in which Type I inflammation promotes a lethal infection with neurological symptoms consistent with cerebral malaria [18], a deficiency in either TLR9 or MYD88 partially protects against lethal disease [26, 28]. However, in contrast to findings with TLR9- and MYD88-deficient mice, in the only study to examine the role of TLR7-deficiency in cerebral malaria, TLR7 was found to be irrelevant for precipitation of fatal disease [26]. This study was performed using small cohorts of mice in an experimental design intended to screen several candidate strains, which allowed for the possibility that a role for TLR7 in cerebral malaria may have been overlooked. To more thoroughly interrogate the role of TLR7 signalling in experimental cerebral malaria in this present study, large sample groups and quantitative measures of immune activation were employed. The current findings demonstrate that the absence of TLR7 during experimental cerebral malaria shifts the balance of cytokines towards an anti-inflammatory state and confers protection from cerebral malaria lethality.

## Methods

### Ethics statement

All mouse work was conducted with the approval of the University of California, San Francisco (UCSF) Institutional Animal Care and Use Committee in strict accordance with the guidelines of the Office of Laboratory Animal Welfare.

### Mice

The following mouse strains were used in this study: C57BL/6 (Jackson Laboratories or the National Cancer Institute); *Tlr7*^−/−^ (Jackson Laboratories); *Tlr9*^−/−^ (R Medzhitov, Yale University; with permission from S Akira, Osaka University, and T Taniguchi, University of Tokyo); *Myd88*^−/−^ (J Cox, UCSF; with permission from S Akira). *Tlr7*^*-/-*^*Tlr9*^*-/-*^double knockout mice were bred in house. All strains were confirmed to have a C57BL/6 background of greater than 95% by microsatellite genotyping.

### Parasites

All experiments were performed using *P. berghei* strain ANKA (MRA-311) parasites, which were obtained from the MR4 stock centre and maintained in C57BL/6 mice. Blood was harvested by cardiac puncture from an infected mouse on day 5 of infection, and 10^6^ infected erythrocytes were introduced into a new mouse by intraperitoneal injection in 100 μl of Alsever’s solution. All infections were initiated at 14.00 hours. For cytokine analysis, blood was harvested by submandibular blood collection or cardiac puncture into K_2_EDTA for plasma isolation.

### Infections

Infections were initiated as described above. Survival and signs of cerebral malaria were monitored daily, and twice daily during the peak of lethality (days 6 through 12). Animals that showed neurological symptoms, such as convulsions, ataxia or paralysis, or that died on or before day 12 post-infection, were considered to have cerebral malaria as previously described [26]. Parasitaemia (percentage of parasite-infected erythrocytes) was monitored daily by Giemsa-stained thin film blood smears. The significance of parasitaemia courses was assessed by the Mann–Whitney U test (α = 0.05). The significance of survival courses was assessed by comparing Kaplan-Meier curves using the log rank (Mantel-Cox) test (α = 0.05).

### Cytokine detection

All plasma cytokines were measured by multiplexed cytometric bead immunoassay (Millipore) as per manufacturer instructions and detected on a MAGPIX (Luminex). Cytokine level significance was assessed using a Mann–Whitney U test (α = 0.05).

## Results

### Loss of TLR7 confers partial resistance to cerebral malaria lethality

**Figure 1:**
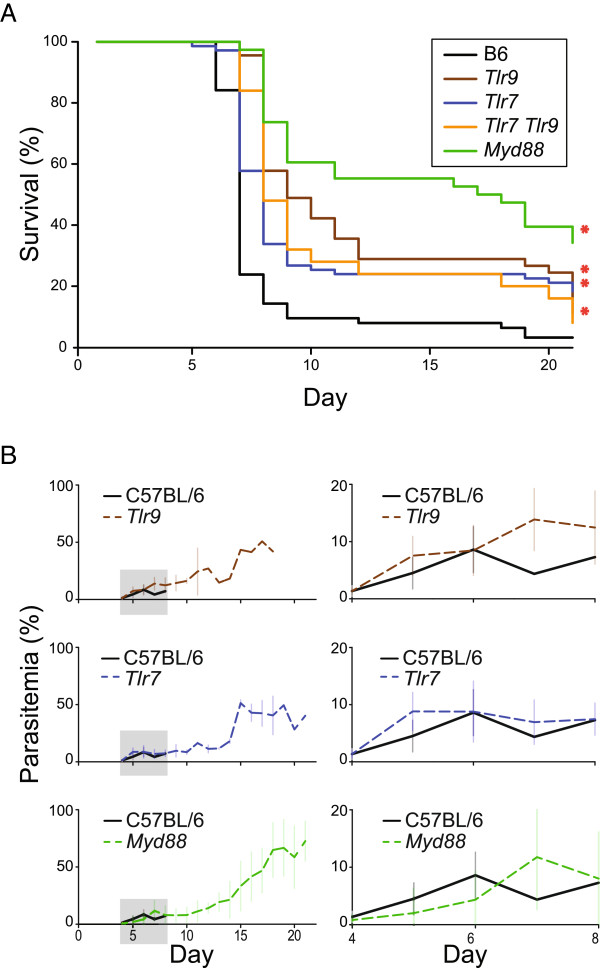
**Survival of TLR-deficient mice during cerebral malaria. (A)** C57BL/6 (B6), *Tlr7* ^-/-^, *Tlr9* ^-/-^ *, Tlr7* ^-/-^ *Tlr9* ^-/-^ *,* and *Myd88* ^-/-^ mice were challenged with 10^6^ *P. berghei* erythrocytes and monitored daily for survival (n = 63 B6, n = 71 *Tlr7* ^*-/-*^, n = 45 *Tlr9* ^−/−^, n = 25 *Tlr7* ^*-/-*^ *Tlr9* ^*-/-*^ *,* n = 38 *Myd88* ^*−/−*^). *, p < 0.05 (log rank [Mantel-Cox] test). Data were pooled from six experiments. **(B)** Infected C57Bl/6, *Tlr7* ^−/−^, *Tlr9* ^*-/-*^ *,* and *Myd88* ^*−/−*^ mice were monitored for parasitaemia. Right, zoomed-in plot of region contained within gray box on left. (n = 25 B6, n = 25 *Tlr7* ^*-/-*^, n = 16 *Tlr9* ^*−/−*^; n = 21 *Myd88* ^*−/−*^). Data were pooled from three independent experiments.

Given the reduced levels of inflammatory cytokines observed in *Tlr7*^-/-^ mice in response to several *Plasmodium sp*. in a previous work [41], it was hypothesized that TLR7 might play a role in the pathology of *P. berghei* cerebral malaria that was previously undetected. To test this, the survival of TLR7-deficient mice, as well as *Tlr9*^*-/-*^*, Tlr7*^*-/-*^*Tlr9*^*-/-*^*,* and *Myd88*^*-/-*^ mice, infected with *P. berghei* parasites was monitored. As previously reported [26, 28], mice deficient in either TLR9 or MYD88 were partially protected from lethal infection with *P. berghei* when compared with wild-type mice, with *Tlr9*^*-/-*^ mice less well protected than *Myd88*^-/-^ mice (29% escape and 58% escape, respectively, with escape defined as survival past day 12 of infection; Figure 1A). These findings are similar to a previous report [28] in which the proportion of *Myd88*^*-/-*^ mice that escaped from cerebral malaria was 1.5 times greater than the proportion of *Tlr9*^*-/-*^ mice, suggesting the existence of additional MYD88-dependent sensors that promote cerebral malaria [26, 27, 29]. In support of the above hypothesis, *Tlr7*^-/-^ mice were partially protected from lethality with approximately 24% escaping from cerebral malaria, as compared to 8% of wild-type mice, a difference that was detectable using large sample sizes (n = 71 for *Tlr7*^*-/-*^ mice). Because TLR7 and TLR9 share the common signalling adapter MYD88, the possibility of a genetic interaction between these sensors was tested by generating mice lacking both sensors. As expected, *Tlr7*^-/-^*Tlr9*^-/-^ mice demonstrated improved survival as compared to wild-type mice, with 24% escaping cerebral malaria. Notably, they were no more protected than mice with either deficiency alone and were also not as well protected as *Myd88*^*-/-*^ mice. There was no significant difference in survival between females and males of any given genotype (log rank [Mantel-Cox] test; α = 0.05). Additionally, protected mice did not show symptoms of cerebral malaria, such as ataxia, hemi- or paraplegia, seizures or coma. This protection was not due to improved parasite restriction; all animals eventually succumbed to hyperparasitaemia after the initial escape from cerebral malaria, as observed in other immunodeficient mice [26, 47] (Figure 1B). The simplest interpretation of these data is that TLR7 and TLR9 synergistically signal to promote cerebral malaria, with both sensors being required for full elaboration of lethal pathology (and conversely, loss of both sensors not conferring more protection than loss of either sensor alone). In addition, it is likely that other MYD88-dependent, but TLR7- and TLR9-independent, mechanisms also promote pathology.

In contrast to the current findings, *Tlr7*^*-/y*^ mice were not found to be protected from cerebral malaria in the single study that has previously examined *P. berghei* infection in TLR7-deficient mice [26]. In the current study, the experimental hazard ratio between B6 and *Tlr7*^*-/-*^ mice was determined to be 2.847, with a probability of 0.888 that a subject of either genotype would succumb to cerebral malaria by the end of the experiment. Based on these numbers, the sample size of five in each group used in the previous study would only provide a statistical power of 0.26 (α = 0.05). In order to detect the difference observed in this study, a minimum sample size of 33 mice is necessary [48]. Based on these calculations, it is possible that the contribution of TLR7 to *P. berghei* pathogenesis was overlooked in the previous work.

**Figure 2:**
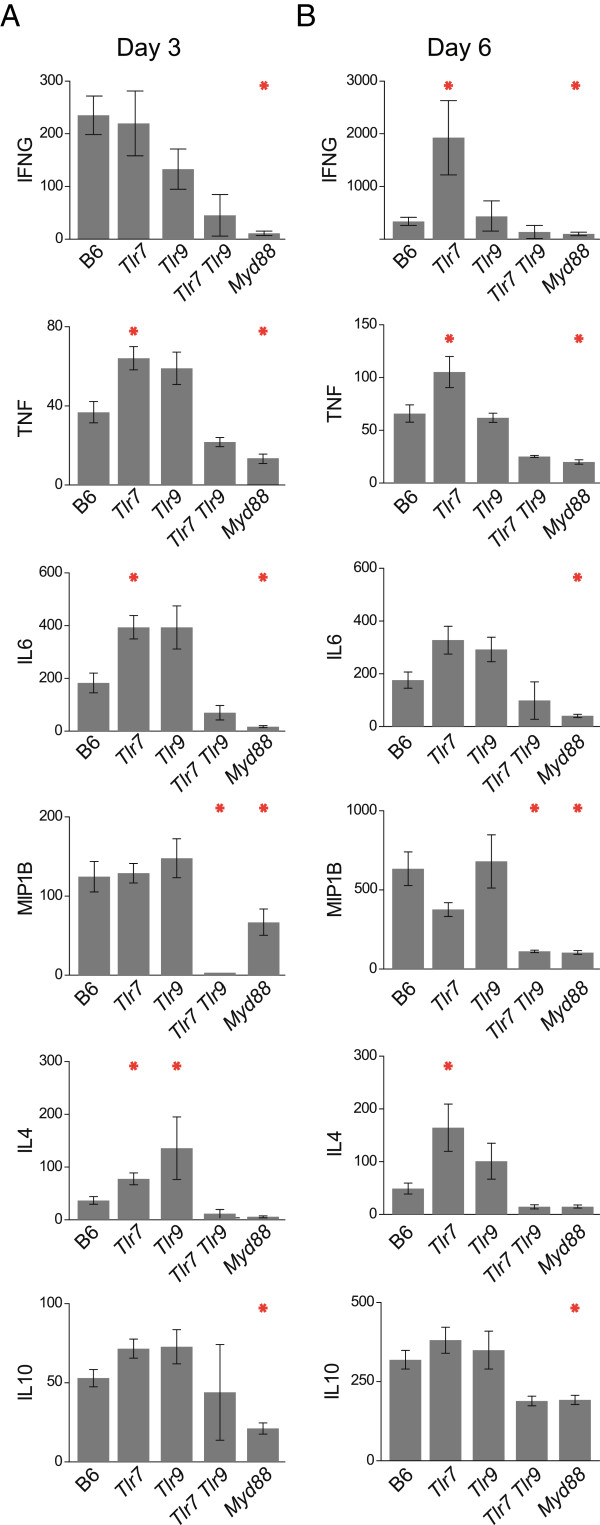
**TLR-dependence of cytokine production during** ***Plasmodium berghei*** **infection. (A, B)** Levels of IFNG, IL6, IL4, TNF, MIP1B, and IL10 were measured by cytometric bead array in plasma collected from mice of the indicated genotypes three days **(A)** or six days **(B)** after infection with 10^6^ *P. berghei*-infected erythrocytes. All units are pg/mL. Means with SE are shown. *, p < 0.05 (Mann-Whitney) (n = 30 B6, n = 22 *Tlr7* ^*-/-*^, n = 11 *Tlr9* ^*−/−*^, n = 3 *Tlr7* ^*-/-*^ *Tlr9* ^*-/-*^, n = 15 *Myd88* ^*−/−*^). Data were pooled from three independent experiments.

To better understand the inflammatory response as related to lethality of *P. berghei* infection in *Tlr7*^-/-^, *Tlr9*^-/-^, *Tlr7*^-/-^*Tlr9*^-/-^, and *Myd88*^*-/-*^ mice, the levels of cytokines that have previously been associated with susceptibility to cerebral malaria [21, 49–52] were measured at three days post-infection, which is at the onset of parasite patency, and six days post-infection, which is approximately 12 to 24 hours before the onset of neurological symptoms in wild-type mice. A subset of both pro-inflammatory cytokines (IFNG, TNF, macrophage inflammatory protein 1 beta [MIP1B], and IL6) and anti-inflammatory cytokines (IL10 and IL4) was found to be dysregulated at various points in the absence of TLR7 and/or TLR9 signalling (Figure 2A and 2B).

Specifically, at both days 3 and 6 post-infection, IFNG, IL6, TNF, MIP1B and IL10 were all significantly diminished in MYD88-deficient mice as compared to wild-type mice. MIP1B was also significantly diminished in *Tlr7*^*-/-*^*Tlr9*^*-/-*^ mice on both days, as compared to wild-type mice. All of the other cytokines also followed the same trend of being reduced in *Tlr7*^*-/-*^*Tlr9*^*-/-*^ as compared to wild-type mice, although they did not reach statistical significance. In contrast, none of these cytokines were significantly diminished in mice singly deficient in either TLR7 or TLR9. In fact, many cytokines were produced at higher levels in *Tlr7*^*-/-*^ mice, including TNF, IFNG, and IL6. *Tlr9*^*-/-*^ mice appeared to follow similar trends for these cytokines, but did not reach significance. These observations are similar to those from early *P. chabaudi* infection [41], wherein mice lacking TLR9 overproduce cytokines; this increase might be a result of decreased competition for endosomal trafficking between TLR7 and TLR9 [53]. Additionally, IL4 was increased in both *Tlr7*^*-/-*^ and *Tlr9*^*-/-*^ mice on day 3, as well as in *Tlr7*^*-/-*^ mice on day 6. Importantly, unlike the synergistic interaction between TLR7 and TLR9 suggested by the survival data, these results are consistent with a model in which TLR7 and TLR9 signal redundantly through MYD88 to promote cytokine production during experimental cerebral malaria.

**Figure 3:**
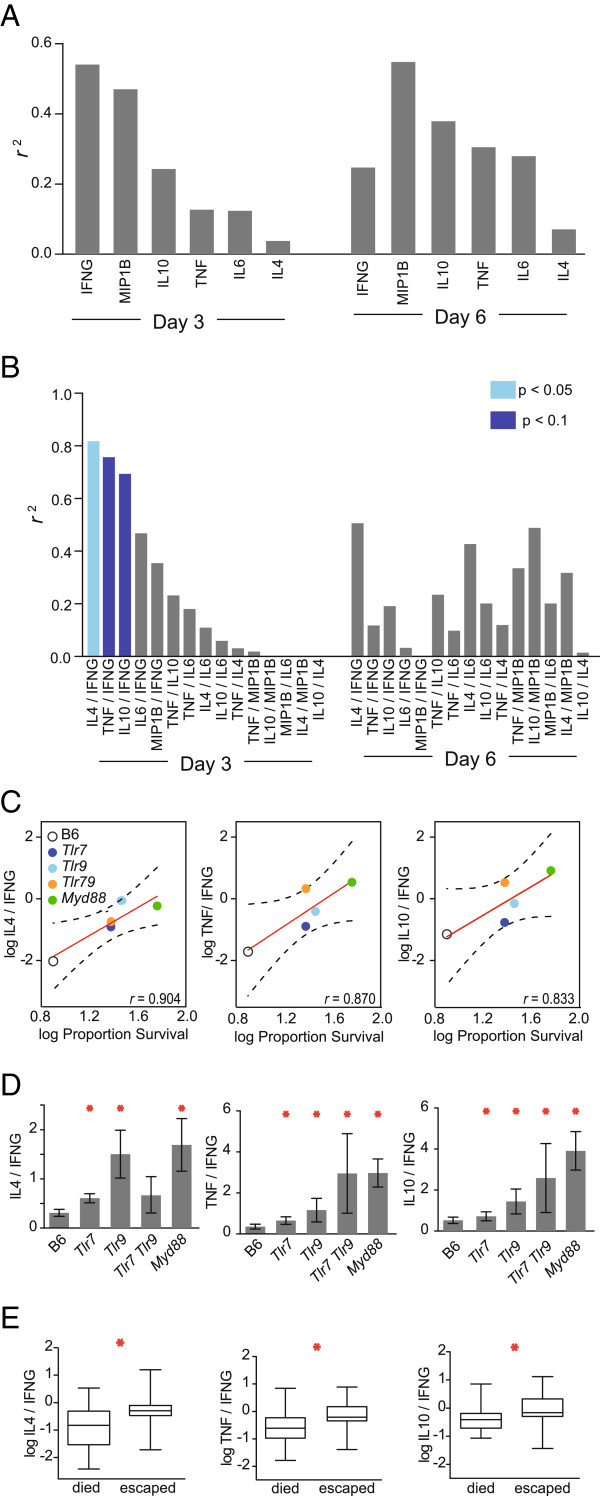
**Association of cytokine ratios with outcomes of experimental cerebral malaria. (A)** For each genotype (B6, *Tlr7* ^*-/-*^, *Tlr9* ^*-/-*^, *Tlr7* ^*-/-*^ *Tlr9* ^*-/-*^ *,* and *Myd88* ^*-/-*^) a Pearson’s product moment correlation was used to determine the relationship between the average log_10_ (plasma cytokine concentration + 1) and the log_10_ fraction of each genotype that escaped cerebral malaria (survived until day 12). *r* ^*2*^ values were calculated from a linear regression of the above. **(B)** As above, except using ratios of cytokines. A less stringent statistical threshold (α = 0.1) was tested to account for the propagation of error when calculating ratios. Light blue, p < 0.05; dark blue, p < 0.1 (Pearson’s correlation). **(C)** Significant log_10_ plasma cytokine ratios from **(B)** as a function of average survival for each genotype. The linear regression across genotypes is shown with 95% confidence intervals. **(D)** Significant cytokine ratios from **(B)** by genotype. Errors represent SE and all statistical comparisons are to B6 mice. *, p < 0.05 (Mann–Whitney). **(E)** Box plots of significantly correlated, log_10_ cytokine ratios for all mice, grouped by escape from cerebral malaria (survival past day 12). *, p < 0.05 (Mann–Whitney).

To assess the relative contributions of each cytokine to the pathogenesis of cerebral malaria, the average plasma levels of each cytokine in each genotype were examined for correlation with the percentage of that genotype that escaped from cerebral malaria. Consistent with the discrepancy between survival and cytokine production, none of the correlations reached a p value of less than 0.1, although *r*^2^ values for IFNG and MIP1B were above 0.5 (days 3 and 6, respectively; Figure 3A). These modest correlations led to the consideration of alternative explanations for the differential survival. Other studies have found that the ratio of certain Type I (*i.e.*, pro-inflammatory) to Type II (anti-inflammatory) cytokines is more strongly associated with severe malaria disease than any single cytokine [54, 55]. Given the established role of pro-inflammatory cytokines in promoting experimental cerebral malaria and anti-inflammatory cytokines in suppressing lethal pathology [18, 19, 22, 47], it was hypothesized that the ratio of such cytokines to one another would be more strongly correlated with survival than the correlation observed for any individual cytokine. Because calculation of ratios results in propagation of error, a less stringent alpha (α = 0.1) was used to test for significant correlations between cytokine ratios and survival. Strikingly, three cytokine log ratios were significantly correlated on day 3 (p < 0.1) (Figure 3B), with correlation coefficients (*r*) of greater than 0.8 (Figure 3C). Additionally, these three ratios, IL4/IFNG, TNF/IFNG, and IL10/IFNG, were all significantly increased in *Tlr7*^*-/-*^*, Tlr9*^*-/-*^*, Tlr7*^*-/-*^*Tlr9*^*-/-*^*,* and *Myd88*^*-/-*^ mice as compared to B6 on day 3 (with the exception of IL4/IFNG in *Tlr7*^*-/-*^*Tlr9*^*-/-*^, which did not reach significance; Figure 3D). Of these three ratios, two represent ratios of anti-inflammatory cytokines (IL4 and IL10) to the pro-inflammatory cytokine, IFNG, suggesting that the balance of these cytokines may be a driver of pathology. The most strongly correlated ratio on day 3, IL4/IFNG, also remained the most strongly correlated on day 6 (just prior to the onset of symptoms in unprotected mice). Notably, these findings might be analogous to those from a study that found the IL4/IFNG ratio to be associated with cerebral malaria in humans [54].To further assess whether these cytokine ratios would delineate susceptibility to cerebral malaria without regard to genetic makeup, mice of all genotypes were grouped by survival status. All three cytokine ratios were significantly elevated in mice that escaped cerebral malaria, as compared to those that succumbed (Figure 3E). These data suggest that TLR signalling in wild-type mice may promote the development of a pathological Type I immune response, whereas the cytokine response in mice deficient in TLR and MYD88 signalling is skewed toward a Type II response that protects against cerebral malaria. Furthermore, regardless of genotype, relative levels of cytokines, particularly the ratio of IL4 to IFNG, are more strongly associated with protection from cerebral malaria than levels of any single cytokine alone.

## Discussion

In this highly powered study, it has been shown that signalling through TLR7 contributes to lethality from cerebral malaria. The loss of both TLR7 and TLR9 signalling leads to cytokine dysregulation during the course of *P. berghei* ANKA infection and protects against symptoms of cerebral malaria. Interestingly, the absence of TLR7 or TLR9 results in overproduction of multiple cytokines, but loss of both TLR7 and TLR9 results in reduced cytokine production (Figure 2). TLR7 and TLR9 have previously been shown to compete via the shared endosomal trafficking molecule, UNC93b1 [53]. It is possible that in mice singly deficient for either TLR7 or TLR9, enhanced signalling by the intact sensor leads to increased cytokine production in response to *P. berghei,* whereas loss of both TLR7 and TLR9 leads to decreased cytokine production. This phenomenon leads to an apparent discrepancy between cytokine production and survival data that can be explained through the consideration of ratios of cytokines to one another, rather than analysis of any single cytokine in isolation.

Additionally, other MYD88-dependent signalling cannot be explained by TLR7 and TLR9 (Figure 1), indicating contributions from other MYD88-dependent sensors. Based on other studies, TLR2 is likely to account for the bulk of the additional MYD88-dependent contribution [26, 29, 33, 39]. Previous studies using the *P. berghei* mouse model of cerebral malaria have shown that multiple pro-inflammatory cytokines, chemokines and leukocyte populations drive the observed rapid lethality [47, 56–58], whereas anti-inflammatory cytokines confer protection from lethality [22, 58, 59]. Consistent with these reports, mice lacking both TLR7 and TLR9, or lacking MYD88, exhibit cytokine profiles skewed toward anti-inflammatory cytokine production and are protected from lethal cerebral malaria. Furthermore, the strong association of cytokine ratios with survival in TLR- and MYD88- deficient mice suggests that protection against pathology may be conferred by the ratio of anti-inflammatory to pro-inflammatory cytokines produced during infection.

Interestingly, one study reported that mice overexpressing TLR7 were also partially protected from cerebral malaria [60]. Although further experiments are needed to reconcile these findings, it is possible that the increased baseline levels of IL10 found in these mice may protect them from subsequent immunopathology. Consistent with this possibility, recombinant IL10 treatment can suppress *P. berghei* lethality [22]. Notably, similar to the protection seen in TLR7-deficient mice, TLR7 overexpression did not have any effect on parasite load [60], indicating that this protection is a consequence of increasing host tolerance to cerebral malaria without affecting host resistance [61].

The mechanistic study of cerebral malaria in humans is difficult, as this disease may be considerably more heterogeneous than is currently appreciated [62, 63]. Given the observation of neurological sequelae following treatment and convalescence in a subset of patients that no longer harbour parasites [64], it is reasonable to expect that some proportion of cases may occur as a result of immunopathology. It is further likely that different sensors contribute to the overall pathology of cerebral malaria differentially in ethnically and genetically diverse individuals, making the identification of all potentially pathogenic molecules desirable. This work identifies TLR7 as yet another molecule involved in the pathological response to *Plasmodium* parasites, and supports the notion that immune responses to *Plasmodium* must be finely tuned to effect parasite clearance while minimizing immunopathology.

## Conclusions

Loss of TLR7 signalling confers partial protection against fatal experimental cerebral malaria, while having no effect on parasite restriction. TLR7 signalling promotes lethal pathology in a manner that is synergistic with TLR9 signalling. The protection conferred by the loss of TLR7 is correlated with a shift towards an anti-inflammatory cytokine profile during *P. berghei* infection.

## Abbreviations

RNA: Ribonucleic acid
TLRs: Toll-like receptors
IFNG: Interferon gamma
TNF: Tumor necrosis factor
IL: Interleukin
MYD88: Myeloid differentiation factor 88
MIP1B: Macrophage inflammatory protein 1 beta.
